# Rethinking the impostor phenomenon: An umbrella review of concept, context and interventions

**DOI:** 10.1111/medu.70076

**Published:** 2025-10-28

**Authors:** Mia Gisselbaek, Mohamed Seyour, Sarah Saxena, Joana Berger‐Estilita, Kori A. LaDonna, Nadia Elia, Georges L. Savoldelli

**Affiliations:** ^1^ Division of Anaesthesiology, Department of Acute Care Medicine Geneva University Hospitals Geneva Switzerland; ^2^ Department of Anaesthesiology, Clinical Pharmacology, Intensive Care and Emergency Medicine Faculty of Medicine, University of Geneva Geneva Switzerland; ^3^ Unit of Development and Research in Medical Education (UDREM), Faculty of Medicine, University of Geneva Geneva Switzerland; ^4^ Division of Pediatric Anesthesia The Montreal Children's Hospital, McGill University Health Center Montreal Quebec Canada; ^5^ Department of Anesthesiology and Perioperative Care Hôpital Universitaire de Bruxelles (H.U.B.), Université Libre de Bruxelles Brussels Belgium; ^6^ Department of Surgery, UMons, Research Institute for Health Sciences and Technology University of Mons Mons Belgium; ^7^ Department of Anesthesiology Helora Mons Belgium; ^8^ Institute for Medical Education University of Bern Bern Switzerland; ^9^ RISE‐Health, Centre for Health Technology and Services Research Faculty of Medicine, University of Porto Porto Portugal; ^10^ Department of Innovation in Medical Education and Department of Medicine, Faculty of Medicine University of Ottawa Ottawa Canada

## Abstract

**Background:**

Impostor Phenomenon (IP) is a psychological experience characterized by persistent self‐doubt and an inability to internalize achievements, leading to negative emotional and professional consequences. In health professions education (HPE), IP is of particular concern because it has been linked to learner well‐being, professional development and potentially to patient safety. Despite growing interest in IP within health care and academic settings, uncertainties remain about how IP is conceptualized, the contextual factors that shape its occurrence and the effectiveness of interventions to address it. This umbrella review synthesizes existing systematic and scoping reviews to examine (1) IP's conceptualization, (2) the contextual influences associated with its occurrence and (3) the effectiveness of interventions aimed at reducing its impact.

**Methods:**

This umbrella review is reported following the PRISMA‐ScR guidelines. A comprehensive literature search was conducted in Medline, Embase, PubMed, PsycINFO, Scopus, Web of Science, CINAHL, Cochrane Library and ERIC from November 27th to December 4th, 2024. Systematic and scoping reviews on IP and related interventions were included. Methodological quality was assessed using AMSTAR‐2 and the Joanna Briggs Institute (JBI) Critical Appraisal Tool.

**Results:**

Sixteen reviews (eight systematic and eight scoping) covering diverse populations and methodologies were included. The reviews revealed a lack of conceptual consistency and challenges in measuring and comparing IP, with no gold‐standard assessment tool. Reported prevalence varied widely– reflecting conceptual and measurement challenges– with risk factors including perfectionism, marginalized status and hierarchical workplace cultures. Coaching, online self‐study modules and mindfulness‐based interventions showed potential in reducing IP intensity, but the methodological quality of intervention studies was inconsistent, with few high‐quality studies.

**Conclusions:**

Results highlight the need for conceptual refinement, standardized measurement tools and rigorous intervention research on IP. Future studies should focus on deconstructing and reconceptualizing IP, as well as developing multidimensional assessment frameworks and evaluating evidence‐based interventions to improve confidence and competence among individuals experiencing IP.

## Abstract 2

Impostor Phenomenon in #HPE remains conceptually messy and hard to measure. Prevalence varies widely, shaped by context and hierarchy. Interventions show promise—but stronger theory, better tools, and higher‐quality research are urgently needed. #MedEd

## 1 INTRODUCTION

Impostor Phenomenon (IP) is characterized by an individual's inability to internalize their successes, often attributing achievements to external factors like luck or connections, rather than their own abilities or efforts.^1^ However, differing frameworks shape how IP is understood and measured, influencing both research and intervention approaches.^2^, ^3^ Although the concept was originally introduced in psychology, IP has gained particular relevance in health profession education (HPE), where the ability to accurately calibrate confidence is integral to patient safety, professional performance and learner wellbeing.^4^, ^5^ Experiencing IP may undermine medical learners' willingness to seek feedback on their practices, take responsibility and engage in collaborative learning, all of which are central to the development of clinical competence.^6^, ^7^, ^8^ Understanding how IP manifests and how it may be mitigated is therefore paramount to developing safe, resilient and effective health professionals.

Over the past decade, there has been a recent surge in IP literature across a wide range of professional contexts, including nursing, medicine and health profession education (HPE).^9^, ^10^ Many cross‐sectional studies have explored the prevalence and risk factors associated with IP.^4^, ^11^, ^12^, ^13^, ^14^ Research suggests that IP is associated with emotional exhaustion, stress, work–family conflict, burnout and suicidal ideation.^10^, ^15^, ^16^, ^17^ Therefore, some research teams have aimed to address the consequences of IP by conducting specific structured interventions, such as coaching, and investigating effective coping strategies, which are self‐initiated responses, such as seeking peer support.^18^, ^19^

Despite a growing number of studies exploring IP, both within and outside the health care context, studies seem to vary in methodological rigour. Existing systematic and scoping reviews have focused on diverse aspects of IP, covering prevalence, psychological impact and intervention studies and seem to point to inconsistent conceptual frameworks.^20^, ^21^, ^22^, ^23^ This variability, both in methodological approaches and topical focus, has left the evidence base fragmented and difficult to interpret. Furthermore, there remains a lack of clarity on how to best support health care learners experiencing IP. Taken together, these issues highlight the need for an umbrella review that synthesizes systematic and scoping reviews across health care and non‐health care contexts, in order to examine how IP is conceptualized, how contextual factors shape it and what is known about the effectiveness of interventions. Addressing these gaps is critical to creating a shared understanding of IP, developing new measurement tools and designing educational and systemic responses in HPE.

Accordingly, this umbrella review synthesizes the existing systematic and scoping reviews to address three questions:

1. How is IP conceptualized across the literature?
2. What are the contextual factors (e.g. workplace environment, systemic bias) associated with the IP?
3. What interventions are effective for addressing the detrimental implications of IP?

By answering these questions, this review aims to map evidence on IP, evaluate the effectiveness of interventions and coping strategies and summarize the current literature to guide future research and practice.

## 2 METHODS

### 2.1 Study design

This umbrella review synthesizes existing evidence on IP from any systematic and scoping reviews. The reporting of this review adheres to the PRISMA‐ScR (Preferred Reporting Items for Systematic Reviews and Meta‐Analyses Extension for Scoping Reviews) guidelines, and the JBI (Joanna Briggs Institute) methodology was employed to ensure rigour in synthesizing systematic reviews.^24^ The review protocol was registered in PROSPERO (CRD42024614095) on December 7, 2024. As this study was a review of previously published literature, no ethical approval was required.

### 2.2 Eligibility criteria

We included all systematic and scoping reviews focusing on IP. To be eligible, reviews had to report systematic searches of established databases and address prevalence, psychological impact, risk factors, contextual factors or evaluate interventions such as cognitive‐behavioural therapy, coaching, psychoeducation or systemic approaches. We made no restriction on professional contexts, population, ages, language or settings included in the reviews. We did not consider reviews published as grey literature (dissertations, theses and non‐peer‐reviewed sources).

### 2.3 Information sources and search strategy

A comprehensive search strategy supported by the librarian of the Faculty of Medicine of the University of Geneva, Switzerland, was conducted in Medline, Embase, Pubmed, PsycINFO, Scopus, Web of Science, CINAHL, Cochrane Library and ERIC on the 27th November, and updated on the 4 December 2024. The complete search strategy is described in Appendix 1. The two main concepts used in the search strategy were Impostor/Imposter and Review. Reference lists of included reviews were also screened to identify additional reviews reporting on IP.

### 2.4 Selection of source of evidence

The selection process was conducted in two stages. First, titles and abstracts were screened to exclude irrelevant studies. Next, the full texts of potentially eligible studies were assessed against the eligibility criteria. Three independent reviewers conducted the screening (MG, SS and JBE), with discrepancies resolved through discussion. The systematic selection of studies was facilitated using **Rayyan 2024 (Cambridge, USA) for the title and abstract screening. Then**, **full texts were extracted in Zotero 6.0 (Center for History and New Media, George Mason University, USA)** to conduct the full‐text screening for eligibility criteria.

### 2.5 Data charting process and data items

Data extraction followed a standardized approach using an **Excel spreadsheet approach (Microsoft Corporation, Redmond, USA)**. One reviewer (MG) conducted the primary data extraction, and a second reviewer (MS) cross‐checked all the original studies. Disagreements were resolved through discussion. Extracted data followed the JBI Data Extraction Form for Review for Systematic Reviews and Research Syntheses, which includes authors, year, design, sample size, population demographics and review focus.^25^

To assess the efficacy of interventions decreasing the IP experience, a list was made of all the interventions mentioned in the reviews. Therefore, a separate Excel spreadsheet was created to extract the data from the original studies testing an intervention that was included in the selected reviews. Two authors independently extracted the data from all included studies (MG and MS), and disagreement was resolved through discussion. Extracted data included: Name of the first author, year of publication, country of origin, study design, population, findings relating to the review objective (e.g. prevalence/incidence, conceptualization, associations and contextual factors), number of participants, type of intervention, the delivery method, data collection methods and outcomes measured. If available, data on control groups, pre‐post differences and effect sizes were also extracted. To evaluate intervention effectiveness, we aimed to conduct a quantitative assessment based on randomized controlled trials (RCTs), but also considering studies of any designs that included control groups, such as non‐randomized controlled trials, pre‐post intervention studies and observational studies (cohort, cross‐sectional or case–control designs). Only studies employing validated measures such as Clance Impostor Phenomenon Scale (CIPS), Young Impostor Scale (YIS), Harvey Impostor Phenomenon Scale (HIPS) or Leary Impostor Scale (LIS) were considered in the quantitative assessment.

### 2.6 Critical appraisal of individual sources of evidence

The methodological quality of systematic reviews was evaluated using AMSTAR 2, a 16‐item checklist designed to assess rigour. Scoping and systematic reviews were assessed for methodological quality using the 11 JBI Scoping Review Critical Appraisal Tool, including clarity of objectives, comprehensiveness of the search strategy, transparency of methods and presentation of results. One point was awarded for each criterion met, with a maximum score of 11. Reviews were categorized as high quality (10–11), moderate quality (7–9) or low quality (≤6) based on their total score. Two reviewers (MG and MS) independently assessed each review, resolving discrepancies through discussion or consultation with a third reviewer.

The quality of evidence from studies included in the embedded sub‐analysis of interventions was assessed using the GRADE framework.

### 2.7 Data analysis and synthesis of results

Qualitative synthesis summarized conceptualization, contextual factors and interventions across all included studies. MAXQDA 2024 (VERBI Software, Berlin, Germany) supported coding and analysis of intervention types. Due to a lack of sufficient high‐quality RCTs, meta‐analysis was not feasible. However, evidence quality was categorized using the GRADE framework to provide a structured assessment of recommendations and confidence in findings.

### 2.8 Research team and reflexivity

The review team brought together individuals with diverse disciplinary and professional backgrounds in health professions education. MG is a Ph.D. student in medical education, focusing her doctoral work on IP. MS is a trainee interested in medical education. SS is a senior clinician and researcher, with an interest in IP. The team also included two senior medical education researchers with expertise in quantitative and qualitative methodologies (JBE and GS), as well as NE, a senior researcher with expertise in systematic searches and meta‐analysis. To ensure the accuracy and comprehensiveness of the search strategy, we consulted a medical librarian who helped design and execute the database searches. KAL was consulted as a content expert to check resonance and consider implications for practice. This multidisciplinary composition strengthened the rigour of the review by allowing for complementary expertise and reflexive interpretation.

## 3 RESULTS

### 3.1 Selection of sources of evidence

**FIGURE 1:**
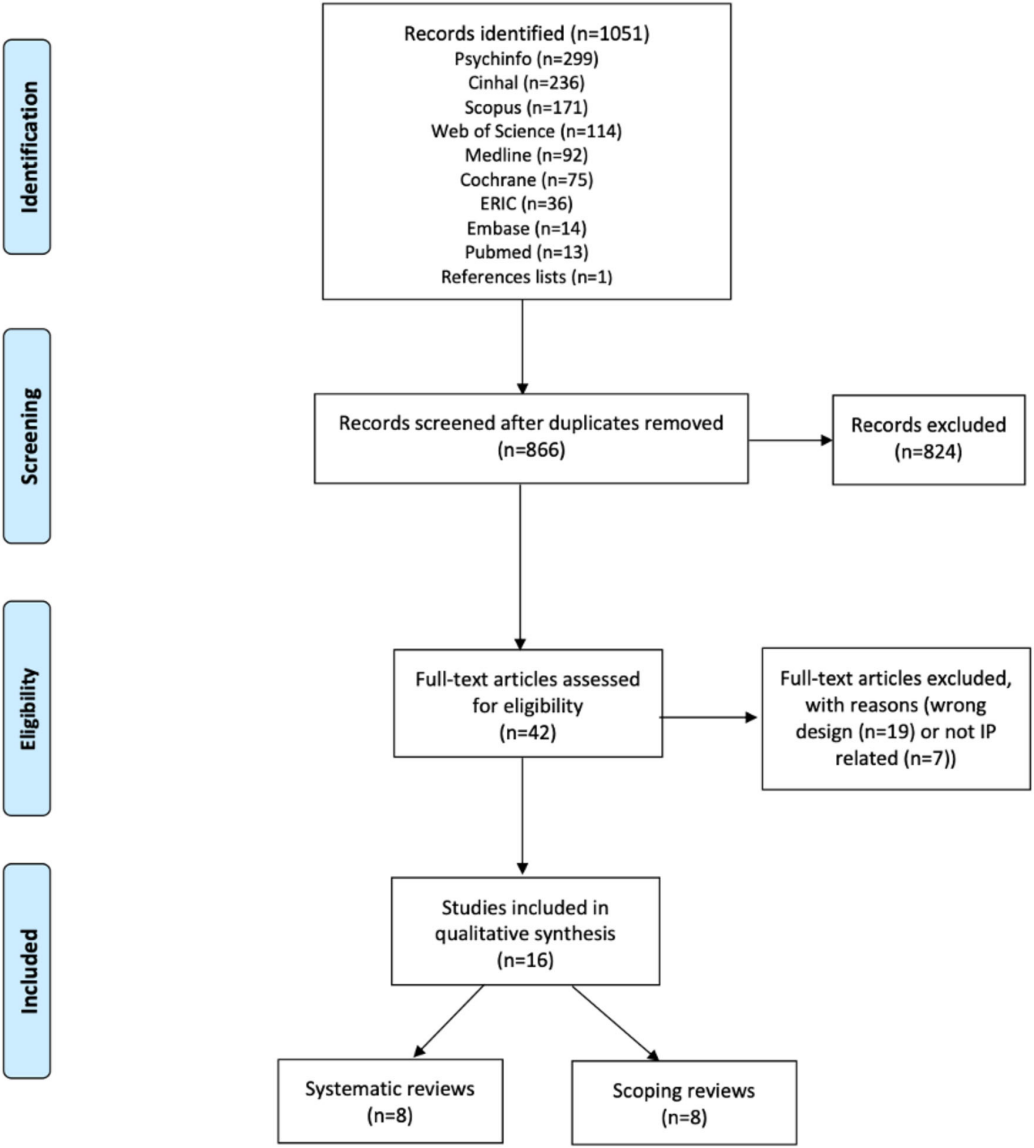
Prisma flowchart of identified and included studies. [Color figure can be viewed at wileyonlinelibrary.com]

The database and literature search yielded 1051 records (866 records after duplicates were removed). After screening titles and abstracts, we excluded 824 records. Following a full‐text review of the remaining articles (n = 42), 26 were excluded because they did not meet all inclusion criteria (19 did not report systematic searches of the literature and 7 were not IP related). The Prisma flowchart is reported in Figure 1.

A total of 16 studies, eight systematic reviews and eight scoping reviews, published between 2019 and 2024 were included. Authors originated from the United States of America (7), Australia (2), Canada (1), China (1), France (1), Israel (1), Malaysia (1), Taiwan (1) and the United Kingdom (1). Surprisingly, most of the reviews pertained to the health professions. The studies included in the reviews were published between 1978 and 2024. Three reviews focused exclusively on intervention studies, and one described the scales used to measure IP.^20^, ^21^, ^23^, ^26^ Two reviews focused on the gender effect of IP and one on the influence of parental factors.^27^, ^28^, ^29^ The rest of the reviews described IP within specific contexts, including the working environment, higher education (undergraduate, postgraduate and faculty university members), among nurses, physicians, health care educators or post‐secondary students^9^, ^10^, ^11^, ^15^, ^30^, ^31^, ^32^, ^33^, ^34^ No review described specific coping strategies for IP. Table 1 summarizes the included reviews. The complete extraction data can be found in Appendix 2.

**TABLE 1:** Summary of reviews on IP. IP: impostor phenomenon, CIPS: Clance impostor phenomenon scale, YIS: Young impostor scale, HIPS: Harvey impostor phenomenon scale. See Appendix 2, for the full extraction chart.

First author, year	Study design	Review objectives	Number of included studies	Summary of findings	Perspectives	Quality assessment of the studies included in the review	JBI critical appraisal checklist	AMSTAR‐2
Hsu, 2024	Systematic review	Assess the impact of online educational intervention on IP and burnout among medical learners.	6	Studies (including RCTs, mixed‐methods, qualitative and feasibility studies) were of high methodological quality, interventions varied including group coaching, workshops and provision of educational resources. Some studies addressed only burnout interventions. Two RCT suggested reduction in IP after online interventions including coaching and self‐study modules. A reduction of burnout, especially emotional exhaustion was also reported after online interventions using coaching and self‐study modules.	Future research is required to explore the potential in developing online interventions that foster self‐compassion and mental well‐being among medical learners	Use MERSQI (Medical Education Research Study Quality Instrument) criteria and conclude that studies are of high quality, even though there is a heterogeneity of study designs.	High (10/11)	Critically Low quality review
Siddiqui, 2024	Scoping review	Explore educational interventions designed to empower high‐achieving individuals with the tools they need to confront IP	17	Two types of educational interventions: Individual (coaching, reflective journaling) and group‐based (psycho‐therapy, interactive workshops and online training module). *The workshops were the most popular with self‐reflection and group‐guided exercises*	High‐quality, longitudinal studies using objective measures and RCT designs are needed to assess intervention effectiveness. In‐depth qualitative assessments to explore participants' experiences are also recommended.	None	Moderate (9/11)	NA
Para, 2024	Scoping review	Map the range of interventions that have been conducted to address IP among individuals experiencing it in a professional context.	31	The characteristics of the studies, the interventions described and the effects of those interventions identified in the literature are heterogeneous. Two major types of interventions emerge: training and counselling. The effect of the interventions varies according to the evaluation methodology used.	Future research should address the gap between limited scientific studies and abundant lay literature on IP. Rigorous methodologies, including systematic pre‐ and post‐intervention measurements and robust experimental or quasi‐experimental designs, are recommended.	Mention high heterogeneity of study designs and intervention types	Moderate (8/11)	NA
Price, 2024	Systematic review and meta‐analysis	Quantify the gender difference in Impostor phenomenon	115	Women consistently score higher in IP than men (mean effect size, Cohen's *d* 0.27, SD 0.11), with no evidence of diminishing gender differences over time. Gender differences are more pronounced in post‐ and undergraduate students than in professionals, and weaker effect sizes are observed in Asian studies compared to those in the USA and Europe. CIPS and its modified versions show greater gender disparities than the Harvey scale. Although IP is often defined as a multidimensional construct, it is rarely measured as such in studies.	Women have higher IP scores than men, and this does not decrease with time.	Assess the publication bias; Estimate of the standard error of each studies, and calculates a significant excess heterogeneity in the effect size	High (11/11)	Low‐quality review
Yang, 2024	Systematic review	Understand IP in higher education (pregraduate, postgraduate and faculty members)	37	IP is prevalent at all stages of higher education: it is strongly associated with pressure, social anxiety and fear of underperformance or rejection. Both males and females experience IP, though it is reported more frequently or intensely among females. Competition and high‐achievement expectations amplify IP. Marginalized groups are particularly vulnerable to IP.	Future research should aim to better understand the underlying mechanisms and effective strategies for addressing IP in higher education.	None	Low (6/11)	Critically Low quality review
Gullifor, 2023	Systematic review	Synthesize the conception of IP in the literature, map IP trait and state nomological work, gaps in the study of IP	200	IP is often conceptualized as a “phenomenon,” with the Clance and Imes (1978) framework being the most widely used, though many studies lack a clear theoretical basis. IP is primarily treated as a trait‐like, enduring construct, limiting understanding of its dynamic, state‐based nature; most research relies on the CIPS scale. Mediators of state IP include shame and fear of failure, whereas moderators include leadership and organizational structures; outcomes span intrapersonal, interpersonal and organizational domains. IP arises from self‐discrepancy theory, where perceived gaps between “other self‐concept” (others' views of us) and “own self‐concept” (our self‐perception) contribute to feelings of impostorism. The literature highlights gaps in methodological rigour and calls for theory‐driven research, longitudinal designs and better exploration of IP's interpersonal and organizational dimensions.	Future research should apply methodological rigour and use of trait and state theories to guide the conceptualization of IP	None	Moderate (9/11)	Critically Low quality review
Edwards‐Maddox, 2023	Scoping review	Understand IP in the nursing profession and the prevalence and severity of burnout among nurses.	18	IP prevalence in nursing ranges from 36% to 75%, with mean CIPS scores for nursing students between 57 and 60. IP is linked to periods of adjustment, transitioning to practice, clinical experience and increased role responsibilities at higher education levels. Gaps exist in the literature regarding IP in newly registered and licensed nurses. All studies utilized cross‐sectional surveys with the CIPS.	Fostering a supportive culture and a sense of belonging may help retain nurses in the professions. The implementation of wellness intervention may alleviate feelings of impostorism.	None	Moderate (8/11)	NA
Ménard, 2023	Scoping Review	Summarize the experiences of the IP among students in higher education.	60	IP is present among post‐secondary students and linked to significant mental health challenges. Findings on IP's relationship with gender and ethnicity are inconsistent. IP negatively impacts self‐esteem and mental health, highlighting the need for targeted interventions in academic settings. Recommendations include raising awareness, normalizing IP feelings and offering workshops, orientations and practical resources to support students. Early interventions are crucial to mitigate mental health issues and safeguard students' long‐term educational and career outcomes.	IP is linked to mental health challenges (e.g. anxiety, depression, low self‐esteem) and may impact academic outcomes. Evidence on equity issues, such as disproportionate effects on marginalized groups, is mixed. Awareness, normalization and early intervention are critical to addressing IP holistically, supporting mental health and academic success	Mention high heterogeneity of study designs and results	Moderate (8/11)	NA
Yaffe, 2023	Systematic review	Summarize what is known on the familial/parental influence on IP and identify gaps for future research	13	Familial and parental factors, such as rearing styles, attachment styles, maladaptive parenting and achievement orientation, show moderate correlations with IP. Parental factors like low care and overcontrol are linked to impostor feelings in offspring. Psychological variables, such as self‐esteem, are stronger predictors of IP than parental influences. Parental and familial factors play a complex, indirect role in the development of IP rather than directly increasing or decreasing it.	Early intervention targeting family dynamics may help reduce IP. Longitudinal studies starting in adolescence are needed to validate cross‐sectional findings.	None	Low (5/11)	Critically Low quality review
Freeman, 2022	Scoping review	Examine and map the concepts of professional identity and impostor phenomenon in health care educators	8	Health care educators often juggle multiple identities (e.g. social, cultural, gender and religious), with professional identity playing a dominant role. Shifts in professional identity can create inconsistencies with past identities, leading to self‐doubt and heightened impostor experiences. Only one study referenced an educational program using metaphors to address impostor phenomenon (IP), but its impact was not reported. IP remains understudied in the context of professional identity and its transitions.	A paucity of research indicates the potential negative impact of IP on the sense of belonging of health care educators. Future research is needed to explore the lived experiences of IP in health care educators.	Mention high heterogeneity of results	Moderate (8/11)	NA
Judie, 2022	Scoping review	Identify the prevalence of IP occurrence, contributing factors and opportunities for future research among health science students.	16	IP prevalence among health science students ranges from 29.5% to 51%, depending on the screening tool. IP is strongly linked to psychological traits, including anxiety, depression, low self‐esteem, poor self‐efficacy and perfectionism. Intense academic and performance pressures in health science fields contribute to the development of IP.	Further research is needed to explore its prevalence and determinants in diverse populations to inform effective interventions and support strategies.	None	Moderate (9/11)	NA
Peng, 2022	Scoping review	Understand the evidence relating to IP in the nursing field.	11	IP prevalence among nurses ranged from 19.3% to 100%, with the largest study reporting 86%. Gender and race were not significantly linked to IP; however, low self‐confidence and self‐esteem were strongly associated. IP was predominantly conceptualized as a stable trait rather than a state. Recognizing that IP is a common experience was shown to be relieving in two studies. Haney et al. (2018) recommended integrating IP awareness into existing courses, emotional education and mentoring programs.	Future studies should build on this review as a foundation for conducting meaningful research on IP among nursing students and nurses.	None	Moderate (9/11)	NA
Vajapey, 2020	Systematic review	Examine data on disparities between men and women in medicine in terms of self‐confidence, self‐efficacy, IP and other assessment factors.	31	Of 31 studies reviewed, 24 found men self‐reported higher confidence in clinical knowledge, skills, procedural competence and other areas than women. Women in graduate and post‐graduate training rated themselves lower in clinical skills, procedural confidence, communication, leadership preparedness and identification with the doctor role. Women reported higher rates of impostor syndrome, stress and burnout compared to men, however the rates were not reported. No evidence showed objective differences in actual performance or skills between men and women at any training level in medicine.	Further studies with a robust methodology are needed to explore the gender effect on the experience of impostor phenomenon, self‐confidence, self‐efficacy and other self‐assessment factors. Relationship.	Uses the GRADE frameworks and concludes that there is mostly Level IV evidence. Mentions a significant heterogeneity in the study designs and the populations studied.	Moderate (9/11)	Low‐quality review
Bravata, 2019	Systematic review	Evaluate the evidence on the prevalence, predictors, comorbidities and treatment of impostor syndrome.	62	IP literature has increased across time with a recent surge of interest. Most studies are coming from english speaking countries (USA, Canada, UK) and almost all study designs were cross‐sectional survey studies. Different scales were used and different cut‐off within the same scales yielding a high heterogeneity. IP is generally highly prevalent (9 to 82%), varies by gender and ethnicity (minorities and women are more affected by the syndrome). Experience and age seem to protect against the phenomena. IP is associated with anxiety and depression, low self‐esteem and social dysfunction. Treatments are underexplored, requiring more rigorous research.	Future research should test treatments for IP in a rigorous manner.	Mention high heterogeneity of study designs	Low (6/11)	Critically Low quality review
Gottlieb, 2019	Scoping review	Map the literature on IP among practicing physicians and physicians in training and identify gaps requiring future research.	18	IP affects 22%–60% of medical students and 33%–44% of resident physicians. Women, low self‐esteem and hierarchical institutional culture are significant risk factors, whereas social support and personal reflections are protective. IP is linked to higher rates of anxiety, depression and burnout, with women more affected due to unconscious bias and perfectionism. Hierarchical medical education environments exacerbate IP. Recommendations include fostering social support, positive affirmation, shared reflections and implementing both individual strategies and institutional reforms.	Future research should aim to further validate assessment tools and better understand the risk or protective factors, especially among underrepresented minorities.	None	Moderate (8/11)	NA
Mak, 2019	Systematic review	Assess the psychometric properties presented in validation studies of IP measurement scales; ascertain whether a gold standard measure of IP exists.	18	Measures reported were CIPS (11 studies) and HIPS (5 studies), with significant variability in the methodological quality of impostorism validation studies. The IP's conceptualization remains underdeveloped, and current measures reflect a unidimensional framework despite multi‐dimensional definitions. Existing scales provide single scores, lacking subdimension granularity, which limits interpretability and consistency across diverse groups (e.g. age, gender, cultures). Longitudinal research and standardized psychometric reporting are needed to improve validity, reliability, and reproducibility of measures. Enhanced scale development and validation studies are crucial to achieve conceptual clarity and trusted representations of IP.	Future research on IP scales should employ rigorous study designs to ensure validity. Current scales are unidimensional, and future studies should explore incorporating multidimensional constructs. A gold standard needs to be established to strenghten future research.	Use Criteria for adequacy of psychometric properties and scoring system (Terwee) and Standards for educational and psychologic testing (American Educational Research Association) and mentions a high heterogeneity of study designs	High (10/11)	Low‐quality review

### 3.2 Characteristics of sources of evidence

The reviews exhibited substantial heterogeneity in study populations, objectives and methodologies. Studies described health care professionals, students or high‐achieving individuals, and examined IP prevalence, risk factors and consequences.^9^, ^11^, ^15^, ^27^, ^28^, ^29^, ^30^, ^32^, ^34^ There was a consensus across the reviews regarding the paucity of well‐conducted research, with a noted lack of high‐quality intervention studies.

Intervention‐focused reviews primarily assessed coaching programs, online self‐study modules and mindfulness‐based interventions aimed at alleviating IP.^20^, ^21^, ^23^ The heterogeneity and diversity in study populations, scales and methodologies prevented cross‐study comparisons and quantitative synthesis.

### 3.3 Critical appraisal of sources of evidence

The reviews demonstrated significant limitations in the quality of reporting, with inadequate assessment of risk of bias and heterogeneity. The systematic reviews were either “Low” or “Critically Low” in quality according to the AMSTAR 2 instrument (Table 1).^35^ Notably, only four reviews mentioned the quality of the studies included in their review, with only three following specific frameworks to rate the quality of studies.^21^, ^26^, ^27^ When using the JBI checklist, three reviews were deemed low quality, ten of moderate quality and three of high quality. The difference in study ratings across appraisal instruments is primarily due to variations in interpretation and precision. The AMSTAR2 instrument employs multiple levels of assessment, whereas the JBI checklist relies on broader, overarching questions. The scores assessing the quality of the included reviews were primarily lowered due to the absence of a comprehensive search strategy, missing protocols and data extraction methods that did not report cross‐checking.

### 3.4 Results of individual reviews

#### 3.4.1 Nonintervention focused reviews

Several reviews examined the prevalence, risk factors, conceptualization and measurement aspects of IP. A limitation noted across reviews was the inconsistent conceptualization of IP and the absence of a gold‐standard measurement tool.

#### 3.4.2 Lack of conceptual consistency

Reviews have highlighted that IP has been conceptualized in markedly different ways, reflecting an ongoing lack of consensus.^15^, ^26^ Across the included reviews, early studies such as Clance's (1978) or Harvey (1981) were summarized as defining IP either as a multidimensional construct encompassing cognitive, emotional and behavioural elements (e.g. Clance, 1978) or an inability to internalize success despite clear evidence of competence (e.g. Harvey 1981).^1^, ^3^, ^36^ Although this trait‐like framing dominates much of the literature and directs interventions towards the individual, Harvey also noted that impostorism can arise in anyone ‐ not only high‐achievers ‐ facing achievement related‐tasks.

As highlighted by Mak et al.' review, building on previous research, Kolligian and Sternberg (1991) proposed the construct of “perceived fraudulence,” which is a multidimensional concept that includes fraudulent ideation, self‐criticism, achievement pressure and impression management.^26^, ^37^ Unlike earlier definitions, this conceptualization highlights the performative aspects of IP, emphasizing vigilant self‐monitoring and concern with social image. Similarly, Leary et al. (2000) emphasized the paradoxical nature of impostorism, focusing on inauthenticity and the belief that others consistently overestimate one's competence.^38^ Together, these contributions underscore that IP has been framed in various ways, such as multidimensional or unidimensional and dispositional or situational.

More recent work has shifted towards contextual, socially constructed framings. Gullifor et al.' review (2023) critiques the field's overreliance on trait‐based assumptions and proposes a trait–state integrative framework.^15^ They argue that impostor experiences often stem from self‐concept incongruence, or the discrepancy between how individuals perceive their competence and how they believe they are evaluated by others. This model highlights the dynamic interplay between enduring dispositions (trait IP) and situationally triggered fluctuations (state IP).

However, this heterogeneity in conceptualization complicates research synthesis and intervention design. Pathology‐based framings encourage cognitive‐behavioural or therapeutic approaches, whereas contextual framings call for systemic strategies that address institutions, hierarchies and inclusion. Thus, the definition of IP directly shapes measurement choices and intervention priorities.

#### 3.4.3 Challenges in measuring and comparing IP

Although measurement scales are designed to assess a construct, the conceptual flaws and different definitions surrounding IP make it difficult to measure and compare a single coherent entity. The absence of a standardized IP assessment tool significantly limits research. The Harvey impostor phenomenon scale (HIPS, Harvey, 1981) was the first instrument; it is a 14‐item tool that captures the inability to internalize success.^26^ The most widely used scale, the 20‐item Clance impostor phenomenon scale (CIPS, Clance, 1985), expands on this concept to include other features such as self‐doubt, discounting success, perfectionism and fear of evaluation.^39^ However, its reliance on a total score conflicts with its multidimensional nature.^15^, ^26^

The 51‐items Perceived Fraudulence Scale (PFS, Kolligan, 1991) emphasizes the multidimensional nature of IP by considering fraudulent ideation, achievement pressure and impression management, highlighting the importance of self‐presentation and social image.^40^ The seven‐items Leary Impostor Scale (LIS, Leary et al. 2000), on the other hand, adopts a unidimensional focus on feeling like a fraud, particularly in socio‐evaluative contexts.^38^ Finally, despite limited psychometric evaluation, the eight‐items Young Impostor Scale (YIS) has been used in multiple RCTs likely because of its brevity and ease of administration.^21^, ^23^

The variation in definitions, cutoffs and psychometric strength across instruments and their use in published studies further hinder comparability and may explain discrepant findings.^11^ For instance, studies using HIPS found weaker gender associations than those using CIPS.^27^

#### 3.4.4 Prevalence, risk factors and consequences

Reported IP prevalence is highly variable and uninformative, depending on the population and the measurement tool.^9^, ^10^, ^11^, ^33^ For instance, Bravata et al. synthesized 62 studies and found prevalences ranging from 9% to 82%, highly depending on the chosen screening tool and cutoff.^11^ Nevertheless, multiple reviews considered IP as frequently observed among health care professionals, high achievers, students and working individuals.^9^, ^10^, ^11^, ^30^, ^31^, ^32^, ^34^ Most reviews remained vague, noting prevalence without enabling meaningful cross‐study comparisons. Yet, a recurring theme across reviews is that, regardless of its conceptualization, IP appears to be a common human experience rather than a marginal phenomenon.

Nevertheless, some recurring risk factors were cited. Notably, parental influences, achievement‐oriented education, underrepresented minority status and hierarchical institutional cultures.^10^, ^15^, ^28^ Being a woman was also consistently linked to higher prevalences. A meta‐analysis found that women generally score higher on IP measures (mean effect size of Cohen's *d* 0.27, SD 0.11),^27^ and another review linked IP in women to increased stress and burnout.^29^

Despite prevalence variations, studies consistently report IP's negative consequences, including burnout, depression, low self‐esteem, anxiety and reduced career progression.^10^, ^11^, ^29^, ^31^ Multiple reviews underscored these associations, noting that the persistence of negative outcomes across contexts strengthens the case for targeted support.^11^, ^20^, ^21^

Interestingly, while negative consequences are repeatedly documented, no reviews systematically examined potential adaptive or motivational aspects of IP, leaving the possibility of “positive” consequences almost entirely unexplored. This absence reflects the broader pathologizing tendency within the literature.

The following section summarizes intervention studies as it is important to mitigate IP's consequences and to support those most at risk.

#### 3.4.5 Intervention‐focused reviews

Three reviews specifically focused on interventions for mitigating IP. All were published in 2024. One review examined online interventions aimed at reducing burnout and IP, whereas the remaining two focused on IP interventions in general.^20^, ^21^, ^23^

A total of 30 studies evaluating interventions were identified across the reviews and are summarized in Appendix 3. The quality of these studies varied widely, ranging from descriptive analyses without efficacy measures to randomized controlled trials (RCTs) reporting pre‐ and post‐intervention IP severity scores. Across these, only five studies were RCTs. When using the GRADE framework to assess the quality of original studies individually, only three studies were rated as “High Quality.”^18^, ^19^, ^41^ Due to inconsistencies in the conceptualization of IP, study designs, measurement tools and reporting practices, a pooled estimate of intervention efficacy was not feasible (Table 2). Furthermore, many intervention studies only reported level‐one outcomes in the Kirkpatrick model, such as participants' immediate reactions or self‐reported satisfaction, rather than demonstrating measurable changes in learning, behaviour or organizational outcomes.^42^

**TABLE 2:** *Study designs of original intervention studies. The publications references of the studies listed in the Table can be found in Appendix* *3*.

Methods	Design	References
Qualitative	Cross‐sectional survey (feedback analysis)	Carlisle (2018)
	Case report	Clance (1978), Clance (1995), Heinrich (1997), Matthews (1985), O'Connel (2020), DeCandia Vitoria (2020)
	Semi‐structured interviews or focus groups	Mann (2022), Magro (2022) Popovic (2020), Stephens (2022)
	Observations (ethnography)	Aird (2017)
Quantitative	RCT	Fainstad (2024a, 2024b, 2022), Mann (2023), Liu (2023), Zanchetta (2020)
	Cross‐sectional survey	Danhauer (2019), Danilewitz (2018), Ogunyemi (2022)
Mixed‐method	Survey study (open and close questions)	Baumann (2020), Chang (2022), Deshmukh (2022), Gold (2019), Haney (2018), Harte (2018), Hutchins (2021), Metz (2020), Rivera (2021),

Of the intervention strategies studied, “coaching” (both face‐to‐face and online), self‐study modules and structured well‐being workshops most consistently demonstrated reductions in IP scores, either in prospective cohort studies or RCTs (Table 3 summarizes the types of interventions).^18^, ^19^, ^43^, ^44^ The **“Better Together Physician Coaching”** program, a four‐month online initiative to reduce burnout and foster self‐compassion among medical professionals, seemed to have the most robust evidence supporting efficacy.^10^, ^38^, ^39^, ^40^ This program incorporated self‐paced materials, live coaching calls and written coaching, demonstrating significant reductions in burnout, moral injury and IP symptoms. However, these IP outcomes were assessed using the YIS, a poorly validated but brief instrument, raising questions about the robustness of the observed effect. Similarly, online self‐study modules incorporating mindfulness exercises were identified as beneficial in reducing IP scores.^41^ These findings suggest that interventions promoting reflection, peer support and self‐compassion may hold particular promise. Yet, the reliance on under‐validated scales and inconsistent reporting practices undermines confidence in their generalizability. More rigorous trials with consistent IP conceptualizations and reliable measures are needed before recommendations. Specifically, researchers testing interventions should begin with a clear conceptualization. This will provide a rationale for their approach and clarify whether IP is viewed as a pathology, a systemic phenomenon or something else. Notably, all of the tested interventions focused on the individual rather than on changing systemic or contextual factors, therefore pathologizing IP.

**TABLE 3:** Type of interventions studied. Note that some studies may appear several times if they reported on mutliple strategies to reduce IP. *The publications references of the studies listed in the Table can be found in Appendix* *3*.

Delivery method	Type of intervention	Reference
Individual	Coaching	Harte (2018), Magro (2022), Zanchetta (2020)
	Reflection	Aird (2017), Stephens (2022)
	Psychotherapy	Clance (1978), Clance (1995), Matthews (1985)
	Mindfulness	Danilewitz (2018)
	Online self‐study module	Fainstad (2024a), Fainstad (2024b), Fainstad (2022), Mann (2022), Mann (2023), Metz (2019), Liu (2023),
	Narrative therapy	DeCandia Vitoria (2020)
Group	Coaching	Fainstad (2024a), Fainstad (2024b), Fainstad (2022), Mann (2022), Mann (2023), Magro (2022)
	Workshop	Baumann (2020), Carlisle (2018), Danhauer (2019), Deshmukh (2022), Gold (2019), Haney (2018), Heinrich (1997), Hutchins (2021), Ogunyemi (2022), O'connel (2020), Popovic (2020), Rivera (2021), Zanchetta (2020)
	Psychotherapy	Clance (1978), Matthews (1985)

Finally, although coping strategies may include informal, self‐initiated practices such as reframing experiences, seeking mentorship or engaging in peer discussions, none of the reviews systematically described these.^45^ Instead, the evidence base focused exclusively on structured interventions delivered through formal programs.

## 4 DISCUSSION

This review underscores the persistent challenges in conceptualizing, measuring and intervening to address IP. Nearly all reviews report significant heterogeneity in study designs, populations and measurement tools, limiting the ability to draw firm conclusions. The literature is predominantly cross‐sectional and relies on unidimensional scales that lack robust validation.

Despite its widespread use as a construct, IP remains conceptually inconsistent, impeding research progress. Current studies conceptualize IP as either a stable trait, rooted in chronic self‐doubt and perfectionism, or a transient state triggered by specific contexts.^15^, ^26^ Yet, some more recent studies suggest that impostor feelings may also be socially produced, reflecting structural inequities, cultural expectations and organizational hierarchies rather than only individual vulnerability.^46^ This tension between “pathology” and “context” frames IP either as a personal deficit to be managed or as an institutional symptom to be addressed. Rather than fitting into categories, IP may be better understood as a spectrum of experiences that fluctuate in intensity and manifestation depending on individual, interpersonal and contextual factors.^47^ As Hafferty's work on the hidden curriculum reminds us, professional identity in medicine is shaped not only by explicit training, but also by tacit cultural messages and exclusionary norms.^48^ In this sense, impostor feelings may reflect not only individual psychology, but also the covert curricula of hierarchy, perfectionism and bias that permeate HPE. For instance, one study found that while impostor feelings were common among medical students, their impact on identity formation varied based on each student's lived experiences and context.^49^ Recognizing this variability can help advance more nuanced research and intervention strategies that reflect the lived realities of those experiencing IP.

Gullifor et al. (2023) proposed that IP arises from self‐concept incongruence, where an individual's self‐perception misaligns with how they believe others perceive their competence, further complicating measurement and interpretation.^15^ Without precise conceptual clarity, measurement tools will inevitably be imperfect reflections, shaping how we continue to (mis)understand the phenomenon. Mak et al. (2024) highlighted that commonly used scales, such as the CIPS, HIPS and PFS scales, lack conceptual clarity and rigorous validation.^26^ These inconsistencies hamper cross‐study comparability and the development of evidence‐based interventions. Without a clear conceptual foundation, existing measurement tools will remain inadequate, making it difficult to compare findings and develop targeted interventions.^26^ These types of challenges are not unique to IP in medical education. Similar challenges have been noted in the research on reflection and reflection assessment.^50^, ^51^ A lack of conceptual clarity hinders the development of valid measurement tools and limits the design of effective educational interventions.^50^, ^51^ Drawing on these parallels, advancing IP research will require psychometric refinement and a precise theoretical foundation to ensure that interventions meaningfully address the phenomenon.

In addition to these methodological challenges, the term “impostor phenomenon” itself warrants re‐evaluation. Clance and Imes used this term in 1978 to describe women who felt like frauds despite clear evidence of competence.^1^ The term “impostor” is related to the internal experience of fraudulence. However, the word can be misleading as being an “impostor” implies dishonesty rather than inaccurate self‐assessment.

Individuals suffering from IP struggle with a misalignment between self‐perception, internal expectations and external validation.^38^

Over time, and across studies, IP has frequently been used as an umbrella term encompassing a variety of related experiences and constructs, including self‐doubt, low self‐efficacy and perfectionism, without sufficiently differentiating these elements.^1^, ^29^, ^52^, ^53^, ^54^ This conceptual vagueness makes it hard to isolate what IP really refers to. Moreover, the professional and cultural context shapes how IP is experienced. IP within academia may differ from how it manifests in the clinical setting, for instance. As such, each profession's specific values, expectations and pressures can shape how people express self‐doubt or fear of being exposed as a fraud.

Importantly, this contextual dimension helps explain why women and underrepresented minorities consistently report higher IP scores. Impostorism may be less about “feeling fraudulent” and more about being made to feel illegitimate by systemic exclusion. This raises questions: does the construct, as originally defined in 1978, still adequately reflect the experience it seeks to describe?

The Intruder Paradox reflects this dilemma: individuals who feel like outsiders despite evidence of competence may not be “impostors”, but rather victims of rigid social or institutional structures that undermine confidence and belonging. First introduced by LaDonna (2025) in her exploration of workplace discrimination and impostorism, this paradox challenges the assumption that self‐doubt always arises from within.^46^ Instead, it suggests that these feelings often stem from external forces systematically questioning an individual's legitimacy. This phenomenon highlights how exclusionary norms, subtle discrimination and imposed perceptions of incompetence can erode individuals' professional identity, reinforcing a cycle of uncertainty.

Given these complexities, a more useful framework may centre on competence, confidence, self‐assessment and professional identity instead of impostorism. Rather than reinforcing a label that may feel diminishing, research should shift towards understanding how individuals navigate competence‐related anxieties, the illusion of incompetence and the role of social belonging in professional identity.^55^

Moving forward, IP should be contemporarily deconstructed and reconceptualized.

Rather than choosing between individual and systemic framings, the field could benefit from integrative models that address both the personal experience of self‐doubt and the structural contexts that exacerbate it. A clearer theoretical model would allow for the development of new, psychometrically validated assessment tools that better reflect the multidimensional nature of the experience. With improved measurement, future RCTs could more effectively evaluate interventions designed to enhance confidence and professional self‐concept, as well as systematic interventions focusing on cultural changes.

In line with this shift, we propose the concept of “appropriate confidence” as a more constructive framework for medical education. Appropriate confidence is the ability to accurately calibrate one's self‐assessment with actual competence. It avoids the underconfidence associated with the impostor phenomenon and the overconfidence of the Dunning–Kruger effect.^5^ By situating impostor experiences within this broader spectrum of confidence regulation, interventions can foster environments that support accurate self‐assessment, self‐efficacy and belonging, rather than pathologizing individuals. This reframing also directs attention towards systemic strategies, such as faculty development, inclusive assessment practices and attention to the hidden curriculum, while retaining the value of individual approaches, like coaching and mentoring. This dual focus aligns with emerging evidence showing that group coaching improves self‐efficacy, fosters inclusion and enhances resilience.^18^, ^19^, ^43^, ^44^

For educators, this means providing support to individual learners and addressing the hidden curriculum that shapes norms of excellence and belonging. Health professions education can work towards a more inclusive and confidence‐sustaining environment by balancing support for individuals with attention to structural reform.

## 5 LIMITATIONS

This umbrella review has several limitations that must be acknowledged. First, the included reviews exhibited substantial **heterogeneity** in study designs, populations and measurement tools, making direct comparisons difficult. The lack of a **gold‐standard measurement too**l for IP further complicates the synthesis of findings, as different studies used varying scales with inconsistent cutoffs, leading to wide‐ranging prevalence estimates and associations. Second, the **methodological quality** of the included systematic and scoping reviews was generally low. According to the AMSTAR‐2 and JBI appraisal tools, many reviews failed to assess risk of bias, incorporate robust inclusion criteria, or report heterogeneity. Only a minority of studies critically evaluated the quality of the evidence they reviewed, limiting the reliability of their conclusions; hence, the conclusion of this review. Third, **conceptual inconsistencies** in defining IP impact the interpretation of findings. Fourth, most of the reviews were health care and higher‐professions education‐related. This likely reflects an increased interest in systematic and scoping review methodologies from these fields but may have overlooked relevant literature from other domains.

Additionally, the **intervention studies identified within the reviews** varied widely in their methodological rigour and designs, which limited the ability to conclude the effectiveness of interventions. Finally, **publication bias and language bias** may have influenced the findings.^56^ The included reviews primarily focused on English‐language publications, potentially omitting studies in other languages.^57^ The tendency to publish studies with significant findings may have led to overrepresenting certain conclusions while underreporting null or contradictory results.^43^

Despite its limitations, this review provides valuable insights for researchers and educators. By pointing out the conceptual ambiguities, methodological weaknesses and systemic blind spots in the existing IP literature, we provide a roadmap for more rigorous and inclusive future research. For faculty and educational leaders, the findings underscore the dual responsibility of supporting individuals and addressing cultural and structural conditions that foster impostor feelings. Addressing both levels of action may ultimately create environments where confidence, belonging and professional identity can flourish.

By systematically reviewing conceptualizations, contextual factors and interventions, this umbrella review highlights limitations and critical gaps in the literature and the need for a **reconceptualization of IP**, the development of **validated multidimensional measurement tools** and **high‐quality intervention research** to support individuals experiencing impostor‐related distress. Moving past outdated terminology and focusing on mechanisms that influence appropriate confidence and competence, research can provide more insights and practical solutions for medical learners struggling with this scope of experiences.

## AUTHOR CONTRIBUTIONS

*Concept and design:* MG, NE, GLS.

*Acquisition, analysis or interpretation of data:* MG, MS, SS, JBE.

*Drafting of the manuscript:* MG.

*Critical review of the manuscript for important intellectual content:* All authors.

*Administrative, technical or material support:* MG, MS.

*Supervision:* KAL, GLS, NE.

## DECLARATION OF GENERATIVE AI IN SCIENTIFIC WRITING

During the preparation of this work, the authors used ChatGPT 4.0 to improve readability. After using this tool/service, the authors reviewed and edited the content as needed and took full responsibility for the content of the publication.

## Supporting information


**Data S1.** Supporting Information.

**Data S2.** Supporting Information.

**Data S3.** Supporting Information.

## Data Availability

The data that support the findings of this study are available from the corresponding author upon reasonable request.
