# Supplementary material for: Rethinking the impostor phenomenon: An umbrella review of concept, context and interventions
Source: Med Educ. 2025 Oct 28;60(6):607–24. doi: 10.1111/medu.70076 (PMC13129615; doi:10.1111/medu.70076)
Supplement: Supplementary file 1 — Data S1. Supporting Information. [file MEDU-60-607-s002.docx]

# Appendix 1 : Search stategy

*The search strategy was supported by a librarian specialized in systematic reviews at Geneva’s University Library of Medicine*

**The key concepts employed for the design of the search strategy were :**

1. *Review or Systematic review or Scoping review or Narrative review or synthesis or searches*
2. *Impostor phenomenon or Impostor syndrome or imposter syndrome or impost* experience or impost* feelings or impostorim*

**The different search strategies are reported hereafter :**

Pubmed : 27/11/2024

(("imposter syndrome" [Supplementary Concept] OR "imposter experience*"[tiab] OR "imposter feelings"[tiab] OR "imposter phenomenon"[tiab] OR "imposter syndrome"[tiab] OR "imposterism"[tiab] OR "impostor experience*"[tiab] OR "impostor feelings"[tiab] OR "impostor phenomenon"[tiab] OR "impostor syndrome"[tiab])) AND (("Systematic Review"[Publication Type:NoExp] OR "Systematic Reviews as Topic"[mesh:noexp] OR "Cochrane Database Syst Rev"[Journal] OR "Evid Rep Technol Assess (Full Rep)"[jour] OR "Evid Rep Technol Assess (Summ)"[jour] OR "scoping"[TI] OR "systematic"[TI] OR ((("comprehensive analysis" [TIAB:~1] OR "comprehensive review" [TIAB:~1] OR "comprehensively reviewed" [TIAB:~1] OR "literature search" [TIAB:~1] OR "literature searches" [TIAB:~1] OR "scoping search" [TIAB:~1] OR "scoping searches" [TIAB:~1]) NOT "narrative review"[TI]) OR "pooled study" [TIAB:~1] OR "systematic search" [TIAB:~1] OR "systematic searches" [TIAB:~1] OR "systematically searched" [TIAB:~1) AND (databases[TIAB] OR "cinahl" [TIAB] OR "cochrane" [TIAB] OR "embase" [TIAB] OR "psycinfo" [TIAB] OR "pubmed" [TIAB] OR "medline" [TIAB] OR "scopus" [TIAB] OR "web science" [TIAB:~1] OR "bibliographic review" [TIAB:~1] OR "bibliographic reviews" [TIAB:~1] OR "literature review" [TIAB:~1] OR "literature reviews" [TIAB:~1]) OR (("electronic database" [TIAB:~1] OR "electronic databases" [TIAB:~1] OR "databases searched" [TIAB:~3]) AND (eligibility [TIAB] OR excluded [TIAB] OR exclusion [TIAB] OR included [TIAB] OR inclusion [TIAB])) OR ("comparative effectiveness" [TIAB:~1] AND "effectiveness review" [TIAB:~2]) OR ("critical interpretive" [TIAB:~1] AND ("interpretive review" [TIAB:~0] OR "interpretive synthesis" [TIAB:~0])) OR ("diagnostic test" [TIAB:~0] AND ("accuracy review" [TIAB] OR "accuracy reviews" [TIAB] OR "accuracy studies" [TIAB] OR "accuracy study" [TIAB]) AND (meta-analysis [TIAB] OR scoping [TIAB] OR systematic [TIAB])) OR ("evidence assessment" [TIAB] AND GRADE [TIAB]) OR ("evidence gap" [TIAB:~2] AND "gap map" [TIAB:~0]) OR "evidence mapping" [TIAB] OR "evidence review" [TIAB] OR "exploratory review" [TIAB] OR "framework synthesis" [TIAB] OR "mapping review" [TIAB:~1] OR "meta epidemiological" [TIAB] OR "meta ethnographic" [TIAB:~0] OR metaethnographic [TIAB] OR "meta ethnography" [TIAB:~0] OR metaethnography [TIAB] OR "meta interpretation" [TIAB:~1] OR "meta narrative" [TIAB:~1] OR "meta review" [TIAB:~1] OR "meta study" [TIAB:~1] OR "meta synthesis" [TIAB:~0] OR metasynthesis [TIAB] OR "meta summary" [TIAB:~1] OR "meta theory" [TIAB:~1] OR "methodological review" [TIAB:~1] OR "methodology review" [TIAB:~1] OR ("mixed methods" [TIAB:~0] AND "methods review" [TIAB:~1]) OR ("mixed methods" [TIAB:~0] AND "methods synthesis" [TIAB:~1]) OR "narrative synthesis" [TIAB:~1] OR "overview reviews" [TIAB:~4] OR ("PRISMA" [TIAB] AND (guideline [TIAB] OR guidelines [TIAB] OR preferred [TIAB] OR reporting [TIAB] OR requirements [TIAB])) OR "PRISMA-P" [TIAB:~0] OR "prognostic review" [TIAB:~1] OR "psychometric review" [TIAB:~1] OR ("qualitative evidence" [TIAB:~0] AND "evidence synthesis" [TIAB:~0]) OR ("qualitative research" [TIAB:~0] AND "research synthesis" [TIAB:~0]) OR ("rapid evidence" [TIAB:~0] AND "evidence assessment" [TIAB:~0]) OR "rapid realist" [TIAB:~0] OR "rapid review" [TIAB:~1] OR "rapid reviews" [TIAB:~1] OR "realist review" [TIAB:~1] OR ("review economic" [TIAB:~1] AND ("economic evaluation" [TIAB:~1] OR "economic evaluations" [TIAB:~1])) OR "review reviews" [TIAB:~1] OR "realist syntheses" [TIAB:~1] OR "realist synthesis" [TIAB:~1] OR "scoping review" [TIAB:~2] OR "scoping reviews" [TIAB:~2] OR "scoping studies" [TIAB:~2] OR "scoping study" [TIAB:~2] OR "systematic evidence map" [TIAB] OR "systematic mapping" [TIAB:~2] OR "systematic literature" [TIAB:~1] OR "systematic Medline" [TIAB:~2] OR "systematic PubMed" [TIAB:~2] OR "systematic review" [TIAB:~2] OR "systematic reviews" [TIAB:~2] OR "systematical review" [TIAB:~1] OR "systematical reviews" [TIAB:~2] OR "systematically identified" [TIAB:~1] OR "systematically review" [TIAB:~1] OR "systematically reviewed" [TIAB:~1] OR "systematized review" [TIAB:~1] OR "umbrella review" [TIAB:~2] OR "umbrella reviews" [TIAB:~2] OR "meta-analysis as topic"[MESH:NOEXP] OR Meta-Analysis[PT] OR "network meta-analysis"[mesh:noexp] OR "indirect comparison"[TIAB:~1] OR meta analyses[TIAB] OR meta analysis[TIAB] OR meta analytic[TIAB] OR meta analytical[TIAB] OR meta analytics[TIAB] OR meta analyze[TIAB] OR meta analyzed[TIAB] OR metaanalyses[TIAB] OR metaanalysis[TIAB] OR metaanalytic[TIAB] OR metaanalyze[TIAB] OR metaanalyzed[TIAB] OR "network comparison"[TIAB:~1] OR "network meta analyses"[TIAB] OR "network meta analysis"[TIAB] OR "network metaanalyses"[TIAB] OR "network metaanalysis"[TIAB] OR (systematic[tiab] AND (meta regression[TIAB] OR metaregression[TIAB]))))

Embase, 27/11/2024

(('impostor phenomenon'/exp OR 'impost?r experience*':ab,ti,kw OR 'impost?r feeling*':ab,ti,kw OR 'impost?r phenomen*':ab,ti,kw OR 'impost?r syndrom*':ab,ti,kw OR 'impost?rism':ab,ti,kw) AND ('systematic review'/de OR 'systematic review (topic)'/de OR (('comprehensive':ti,ab,kw OR 'mapping':ti,ab,kw OR 'methodology':ti,ab,kw OR 'scoping':ti,ab,kw OR 'systematic':ti,ab,kw) AND ('search':ti,ab,kw OR 'searched':ti,ab,kw OR 'searches':ti,ab,kw OR 'studies':ti,ab,kw) AND ('cinahl':ti,ab,kw OR 'cochrane':ti,ab,kw OR 'embase':ti,ab,kw OR 'psycinfo':ti,ab,kw OR 'pubmed':ti,ab,kw OR 'medline':ti,ab,kw OR 'scopus':ti,ab,kw OR 'web of science':ti,ab,kw OR 'bibliographic review':ti,ab,kw OR 'bibliographic reviews':ti,ab,kw OR 'literature review':ti,ab,kw OR 'literature reviews':ti,ab,kw OR 'literature search':ti,ab,kw OR 'literature searches':ti,ab,kw OR 'qualitative review':ti,ab,kw OR 'qualitative reviews':ti,ab,kw OR 'quantitative review':ti,ab,kw OR 'quantitative reviews':ti,ab,kw)) OR 'comprehensive review':ti,ab,kw OR 'comprehensive reviews':ti,ab,kw OR 'comprehensive search':ti,ab,kw OR 'comprehensive searches':ti,ab,kw OR 'critical review':ti,ab,kw OR 'critical reviews':ti,ab,kw OR (('electronic database':ti,ab,kw OR 'electronic databases':ti,ab,kw OR (databases NEAR/3 searched)) AND (eligibility:ti,ab,kw OR excluded:ti,ab,kw OR exclusion:ti,ab,kw OR included:ti,ab,kw OR inclusion:ti,ab,kw)) OR 'evidence assessment':ti,ab,kw OR 'evidence review':ti,ab,kw OR 'exploratory review':ti,ab,kw OR 'framework synthesis':ti,ab,kw OR 'mapping review':ti,ab,kw OR 'meta-review':ti,ab,kw OR 'meta-synthesis':ti,ab,kw OR 'methodology review':ti,ab,kw OR 'mixed methods review':ti,ab,kw OR 'mixed methods synthesis':ti,ab,kw OR (overview NEAR/4 reviews) OR 'prisma':ab OR ('preferred':ti,ab,kw AND reporting:ti,ab,kw) OR 'prognostic review':ti,ab,kw OR 'psychometric review':ti,ab,kw OR 'rapid evidence assessment':ti,ab,kw OR 'rapid literature review':ti,ab,kw OR 'rapid literature search':ti,ab,kw OR 'rapid realist':ti,ab,kw OR 'rapid review':ti,ab,kw OR 'rapid reviews':ti,ab,kw OR 'realist review':ti,ab,kw OR 'review of reviews':ti,ab,kw OR 'scoping review':ti,ab,kw OR 'scoping reviews':ti,ab,kw OR 'scoping study':ti,ab,kw OR 'systematic evidence map':ti,ab,kw OR 'systematic evidence mapping':ti,ab,kw OR 'systematic literature':ti,ab,kw OR 'systematic medline':ti,ab,kw OR 'systematic pubmed':ti,ab,kw OR 'systematic review':ti,ab,kw OR 'systematic reviews':ti,ab,kw OR 'systematic search':ti,ab,kw OR 'systematic searches':ti,ab,kw OR 'systematical literature review':ti,ab,kw OR 'systematical review':ti,ab,kw OR 'systematical reviews':ti,ab,kw OR 'systematically identified':ti,ab,kw OR 'systematically review':ti,ab,kw OR 'systematically reviewed':ti,ab,kw OR 'umbrella review':ti,ab,kw OR 'umbrella reviews':ti,ab,kw OR '13616137':is OR 'cochrane database of systematic reviews'/jt OR 'meta analysis'/de OR 'network meta-analysis'/de OR 'meta analysis (topic)'/de OR 'meta analyses':ti,ab,kw OR 'meta analysis':ti,ab,kw OR 'meta analytic':ti,ab,kw OR 'meta analytical':ti,ab,kw OR 'meta analytics':ti,ab,kw OR 'meta analyze':ti,ab,kw OR 'meta analyzed':ti,ab,kw OR 'meta regression':ti,ab,kw OR 'metaanalyses':ti,ab,kw OR 'metaanalysis':ti,ab,kw OR 'metaanalytic':ti,ab,kw OR 'metaanalyze':ti,ab,kw OR 'metaanalyzed':ti,ab,kw OR 'metaregression':ti,ab,kw OR 'network meta analyses':ti,ab,kw OR 'network meta analysis':ti,ab,kw OR 'indirect treatment comparison':ti,ab,kw OR (('indirect':ti,ab,kw OR 'indirectly':ti,ab,kw OR 'mixed':ti,ab,kw) AND ('treatment':ti,ab,kw OR 'treatments':ti,ab,kw OR 'intervention':ti,ab,kw OR 'interventions':ti,ab,kw OR 'therapeutic':ti,ab,kw OR 'therapeutics':ti,ab,kw) AND ('comparison':ti,ab,kw OR 'comparisons':ti,ab,kw) AND ('bayesian':ti,ab,kw AND 'statistical':ti,ab,kw OR 'bayesian statistics':ti,ab,kw))))

Web of Science : 27/11/2024

(('impostor phenomenon' OR 'impost?r experience*' OR 'impost?r feeling* OR 'impost?r phenomen*' OR 'impost?r syndrom*' OR 'impost?rism') AND ('systematic review' OR (('comprehensive 'OR 'mapping' OR 'methodology' OR 'scoping' OR 'systematic') AND ('search' OR 'searched' OR 'searches' OR 'studies') AND ('cinahl' OR 'cochrane' OR 'embase' OR 'psycinfo' OR 'pubmed' OR 'medline' OR 'scopus' OR 'web of science' OR 'bibliographic review' OR 'bibliographic reviews' OR 'literature review' OR 'literature reviews' OR 'literature search' OR 'literature searches' OR 'qualitative review' OR 'qualitative reviews' OR 'quantitative review' OR 'quantitative reviews')) OR 'comprehensive review' OR 'comprehensive reviews' OR 'comprehensive search' OR 'comprehensive searches' OR 'critical review' OR 'critical reviews'OR (('electronic database'OR 'electronic databases'OR (databases NEAR/3 searched)) AND (eligibility OR excluded OR exclusion OR included OR inclusion)) OR 'evidence assessment' OR 'evidence review' OR 'exploratory review' OR 'framework synthesis' OR 'mapping review' OR 'meta-review' OR 'meta-synthesis' OR 'methodology review' OR 'mixed methods review' OR 'mixed methods synthesis' OR (overview NEAR/4 reviews) OR 'prisma' OR ('preferred'AND reporting) OR 'prognostic review' OR 'psychometric review' OR 'rapid evidence assessment' OR 'rapid literature review' OR 'rapid literature search' OR 'rapid realist' OR 'rapid review' OR 'rapid reviews' OR 'realist review' OR 'review of reviews' OR 'scoping review' OR 'scoping reviews' OR 'scoping study' OR 'systematic evidence map' OR 'systematic evidence mapping' OR 'systematic literature' OR 'systematic medline' OR 'systematic pubmed' OR 'systematic review' OR 'systematic reviews' OR 'systematic search' OR 'systematic searches' OR 'systematical literature review' OR 'systematical review' OR 'systematical reviews' OR 'systematically identified' OR 'systematically review' OR 'systematically reviewed' OR 'umbrella review' OR 'umbrella reviews' OR 'cochrane database of systematic reviews' OR 'meta analysis' OR 'network meta-analysis' OR 'meta analyses' OR 'meta analytic' OR 'meta analytical' OR 'meta analytics' OR 'meta analyze' OR 'meta analyzed' OR 'meta regression' OR 'metaanalyses' OR 'metaanalysis' OR 'metaanalytic' OR 'metaanalyze' OR 'metaanalyzed' OR 'metaregression' OR 'network meta analyses' OR 'network meta analysis' OR 'indirect treatment comparison' OR (('indirect' OR 'indirectly'OR 'mixed') AND ('treatment' OR 'treatments' OR 'intervention' OR 'interventions' OR 'therapeutic' OR 'therapeutics') AND ('comparison' OR 'comparisons') AND ('bayesian' AND 'statistical' OR 'bayesian statistics'))))

ERIC : 27/11/2024

("impostor phenomenon" OR "impostor syndrome" OR "imposter phenomenon" OR "imposter syndrome" OR "imp?st?" OR "self-doubt" OR "Impostorisme" ) AND ("review" OR "literature review" OR "systematic review" OR "meta-analysis")

Scopus : 27/11/2024

(TITLE-ABS-KEY("impostor phenomenon" OR "impostor syndrome" OR "imposter phenomenon" OR "imposter syndrome" OR "self-doubt")) AND (TITLE-ABS-KEY("review" OR "literature review" OR "systematic review" OR "meta-analysis"))

PsychINFO : 27/11/2024

(DE "Impostor Syndrome" OR DE "Self Concept" OR DE "Professional Identity" OR DE "Self Esteem" OR DE "Perfectionism" OR "impostor phenomenon" OR "impostor syndrome" OR "impostorism" OR "imposter phenomenon" OR "imposter syndrome" OR "self-doubt" OR "feelings of inadequacy" OR "academic impostorism" OR "professional impostorism" OR "self-perception" OR "self-handicapping beliefs" OR "fear of failure" OR "self-efficacy doubts") AND (DE "Literature Review" OR DE "Systematic Review" OR DE "Meta Analysis" OR "review" OR "literature review" OR "systematic review" OR "meta-analysis" OR "evidence synthesis" OR "research review")

CINHAL : 27/11/2024

(MH "Imposter Syndrome" OR "impostor phenomenon" OR "impostor syndrome" OR "imposter phenomenon" OR "imposter syndrome" OR "self-doubt" OR "feelings of inadequacy" OR "academic impostorism" OR "professional impostorism" OR "self-perception" OR "fear of failure" OR "self-efficacy doubts") AND (MH "Review Literature as Topic" OR MH "Meta-Analysis" OR MH "Systematic Review" OR "review" OR "literature review" OR "systematic review" OR "meta-analysis" OR "evidence synthesis" OR "integrative review" OR "scoping review")

Cochrane library : 04/12/2024

("impostor phenomenon" OR "impostor syndrome" OR "imposter phenomenon" OR "imposter syndrome" OR "self-doubt" OR "feelings of inadequacy" OR "academic impostorism" OR "professional impostorism" OR "self-perception" OR "fear of failure" OR "self-efficacy doubts") AND ("review" OR "systematic review" OR "meta-analysis" OR "evidence synthesis" OR "integrative review" OR "scoping review")

Medline : 04/12/2024

(MH "Imposter Syndrome" OR MH "Self Concept" OR OR MH "Self Efficacy" OR MH "Professional Identity" OR MH "Achievement Motivation" OR MH "Perfectionism" OR "impostor phenomenon" OR "impostor syndrome" OR "imposter phenomenon" OR "imposter syndrome" OR "self-doubt" OR "feelings of inadequacy" OR "academic impostorism" OR "professional impostorism" OR "self-perception" OR "fear of failure" OR "self-efficacy doubts")
AND (MH "Review Literature as Topic" OR MH "Meta-Analysis" OR MH "Systematic Review" OR "review" OR "literature review" OR "systematic review" OR "meta-analysis" OR "evidence synthesis" OR "integrative review" OR "scoping review")
